# Cardiac Structure and Function in 8‐ to 12‐Year‐Old Children Following In‐Utero Exposure to Preeclampsia (FINNCARE Study)

**DOI:** 10.1161/JAHA.124.034494

**Published:** 2024-07-16

**Authors:** Michelle Renlund‐Vikström, Tiina J. Jääskeläinen, Anni Kivelä, Seppo Heinonen, Hannele Laivuori, Taisto Sarkola

**Affiliations:** ^1^ Children’s Hospital University of Helsinki and Helsinki University Hospital Helsinki Finland; ^2^ Minerva Foundation Institute for Medical Research Helsinki Finland; ^3^ Medical and Clinical Genetics University of Helsinki and Helsinki University Hospital Helsinki Finland; ^4^ Department of Food and Nutrition University of Helsinki Helsinki Finland; ^5^ Department of Obstetrics and Gynecology Helsinki University Hospital Helsinki Finland; ^6^ Department of Obstetrics and Gynecology Tampere University Hospital and Tampere University, Faculty of Medicine and Health Technology, Tampere Center for Child, Adolescent, and Maternal Health Research Tampere Finland

**Keywords:** adiposity, blood pressure, cardiac function, left atrial volume, left ventricular mass, preeclampsia, Pediatrics, Preeclampsia, Blood Pressure, Echocardiography

## Abstract

**Background:**

We evaluated how elevated blood pressure in children exposed to preeclampsia (PE) impacted on their cardiac structure and function, as well as relations with maternal, gestational, and perinatal factors and child body size and composition.

**Methods and Results:**

A total of 182 PE (46 early‐onset preeclampsia) and 85 unexposed (non‐PE) children were examined in the FINNCARE study 8 to 12 years after the index pregnancy with echocardiography; office, central, and 24‐hour ambulatory blood pressures; and body anthropometrics and composition. PE children had lower right ventricular basal sphericity index (mean difference, −0.26 95% CI, −0.39 to −0.12) and lower mitral lateral *E*′‐wave peak velocity (−1.4 cm/s [95% CI, −2.1 to −0.6]), as well as higher *E* to *E*′ ratio (0.40 [95% CI, 0.15–0.65]) and indexed tricuspid annular plane systolic excursion (0.03 [95% CI, 0.01–0.05]) compared with non‐PE children. These differences were accentuated in early‐onset PE children. Left ventricular mass (LVM) or left atrial volume were not different between PE and non‐PE children. Lean body mass, body fat percentage, and 24‐hour systolic blood pressure were independent predictors of LVM. Lean body mass and body fat percentage were independent predictors of left atrial volume. No significant associations between LVM or left atrial volume and maternal, gestational, or perinatal parameters were found.

**Conclusions:**

Preadolescent PE children display a more globular‐shaped right ventricle with higher longitudinal systolic displacement as well as mildly altered diastolic indices, with the alterations being pronounced in early‐onset preeclampsia. Lean body mass and adiposity are independently related with LVM and left atrial volume, and systolic blood pressure with LVM in both PE and non‐PE children. These unfavorable associations indicate remodeling of cardiac structure in young children also reflected in mild functional changes in PE children.

**Registration:**

URL: https://www.clinicaltrials.gov; unique identifier: NCT04676295.

**Nonstandard Abbreviations and Acronyms**
FINNPEC: The Finnish Genetics of Pre‐eclampsia Consortium
LAV: left atrial volume
LBM: lean body mass
LVM: left ventricular mass
PE: preeclampsia‐exposed

**Clinical Perspective**

**What Is New?**

- Preadolescent children exposed to preeclampsia in utero show a more globular‐shaped right ventricle and mildly altered left ventricle diastolic indices compared with a control group.
- The cardiac structural and functional findings are accentuated in the early‐onset children exposed to early‐nset preeclampsia.

**What Are the Clinical Implications?**

- Preeclampsia exposure in utero seems to impact children's cardiovascular risk profile, including cardiac structure and function, already in preadolescence; this supports the importance of accounting for perinatal history when assessing children's cardiovascular health in the clinic.
- Early screening of blood pressure and adiposity are important in children following preeclampsia exposure.
- Our data support primarily using lean body mass to adjust left ventricular mass and left atrial volume for body size in preadolescent children.

High blood pressure (BP) is well known to be linked with increased left ventricular mass (LVM), and LVM has been coupled with elevated risk of overall cardiovascular disease (CVD) death and morbidity in adulthood.^1^, ^2^ Hypertension, increased body mass index (BMI), and male sex are associated with increased LVM in preadolescent children and adolescents.^3^, ^4^ Other studies have also shown childhood and young adulthood obesity impacting LVM^5^, ^6^ and overall cardiac structure and function.^7^, ^8^

Preeclampsia is a hypertensive disorder of pregnancy and is diagnosed in up to 10% of all pregnancies.^9^ A recent cohort study found no differences in cardiac structure or function in 2 to 10‐year‐old children exposed to preeclampsia (PE) (including early‐ and late‐onset preeclampsia) compared with controls.^10^ Limited impact of preeclampsia on offspring cardiac structure and function in childhood was also noted in a previous small sample‐size study.^11^ Later during adolescence, prior fetal exposure to preeclampsia has, nevertheless, been associated with changes in cardiac structure, and child cardiac parameters seem to be influenced by maternal increase in systolic BP (SBP) during early pregnancy.^12^ Furthermore, preeclampsia is associated with increased risk of perinatal death and morbidity (including prematurity and low birth weight).^13^ Preterm birth has been coupled with structural changes in the myocardium^14^ and impaired fetal growth with cardiovascular changes already in the fetus.^15^ Prematurity is associated with increased LVM and impaired cardiac function in adulthood, and preeclampsia further predisposes to reduced systolic function.^16^ Young children born small for gestational age (SGA) or large for gestational age have shown cardiac structure to mainly be predicted by lean body mass (LBM), with limited alterations in cardiac diastolic function.^17^ Altogether, there are limited studies investigating the cardiac structure and function of PE children.

We have recently reported increased BP (office, 24‐hour, and central) and arterial stiffness (by pulse wave velocity analysis) in preadolescent PE children compared with age‐matched unexposed (non‐PE) controls,^18^ as well as reported associations between BP, adiposity, and arterial health in PE and non‐PE children.^19^ Our hypothesis was that PE children's cardiac structure and function are influenced by BP and body composition. We therefore cross‐sectionally evaluated relations between BP and cardiac structure and function in PE children 8 to 12 years after birth compared with age‐matched non‐PE children. Furthermore, we assessed associations with maternal, gestational, and perinatal factors, as well as postnatal growth and child body anthropometrics and composition at follow‐up.

## Methods

### Study Design, Sample, and Setting

We report on 182 PE and 85 non‐PE children examined 8 to 12 years following index pregnancy at the Clinical Trial Unit at Children's Hospital in Helsinki, Finland, from June 2019 to June 2022. This article is part of the FINNCARE (NCT04676295) study,^20^ and CVD risk profiles were evaluated in a prospective cohort study setting for PE and non‐PE families living in the Hospital district of Helsinki and Uusimaa. Study families were recalled from the FINNPEC (Finnish Genetics of Pre‐eclampsia Consortium) multicenter study cohort,^21^ and on the basis of inclusion and exclusion criteria, 366 of 471 families experiencing preeclampsia and 233 of 307 families not experiencing preeclampsia living in the Hospital district of Helsinki and Uusimaa were contacted. The FINNCARE study's exclusion criteria included mothers' inability to communicate in Finnish, ongoing pregnancy or lactation, and multiple pregnancy. Exclusion criteria for mothers without preeclampsia were additionally preeclampsia, gestational hypertension, chronic hypertension, gestational diabetes or diabetes during or after the index pregnancy. Definition for preeclampsia in the FINNPEC cohort was hypertension and proteinuria after 20 gestational weeks (SBP ≥140 mm Hg or diastolic BP ≥90 mm Hg, and urinary excretion of ≥0.3 g protein in a 24‐hour specimen, or 0.3 g/L, or 2 ≥1+readings on dipstick).

This article provides a cross‐sectional evaluation of the children's echocardiography parameters before randomization into intervention and control groups. The Ethics Committee of the Hospital District of Helsinki and Uusimaa approved the FINNCARE study protocol in December 2018 (HUS/3347/2018). All study participants signed an informed consent. Participating (N=192) and nonparticipating (N=118) mothers with preeclampsia from the Hospital district of Helsinki and Uusimaa FINNPEC cohort were examined for potential recruitment bias (data missing for 56 nonparticipating mothers with preeclampsia), but did not differ in maternal, gestational, and child perinatal characteristics (results not shown).

The authors declare that all supporting data are available within the article (and its online supplementary files).

### Echocardiography

Transthoracic 2‐dimensional echocardiography (Vivid 7, General Electric Medical Systems, Horten, Norway) was performed by an experienced pediatric cardiologist. Stored images and cine clips were analyzed offline using the IntelliSpace Cardiovascular 5.2 software (Philips), and strain assessments were performed using EchoPAC software (Viewpoint 6). Images were acquired and analyzed according to guidelines.^22^ Frame rates were between 40 and 70 Hz during image clip acquisition. Dimensions of the aortic arch, proximal aorta, and aortic valves as well as pulmonary valves were measured in systole. Inferior vena cava diameter respiratory change in percentage was calculated. Aortic pulse wave velocity was calculated as arterial distance divided by transit time. Arterial distance was calculated by subtracting 5 cm from right carotid–femoral direct distance assessed with a tape measure. Transit times were assessed at the aortic isthmus and femoral artery using pulsed wave Doppler in relation with the R‐wave on ECG. Abdominal aortic compliance and abdominal aortic artery stiffness index were calculated from the following formulas:

$$\begin{array}{c} \text{ABDAOCOMP}\\ =1000\times \left(\frac{\text{ABDAOMAX}-\text{ABDAOMIN}}{\text{ABDAOMIN}}\right)/\left(\mathrm{SBP}-\mathrm{DBP}\right) \end{array}$$

$$\text{ABDAOSTIF}=\ln\left(\frac{\mathrm{SBP}}{\mathrm{DBP}}\right)/\left(\frac{\text{ABDAOMAX}-\text{ABDAOMIN}}{\text{ABDAOMIN}}\right)$$

where ABDAOMAX is the abdominal aorta lumen dimension in end‐diastole, ABDAOMIN the abdominal aorta lumen dimension in peak‐systole, SBP the office systolic blood pressure, and DBP the office diastolic blood pressure.

We used Devereux's formula to calculate LVM from left ventricular dimensions obtained from the parasternal short‐axis view^23^ and LVM *Z* scores were generated for sex and LBM.^24^ LVM was also assessed indexed to height^2.7^.^25^ Left ventricular end‐diastolic and end‐systolic volumes were assessed with the Simpson biplane method from standard 4‐ and 2‐chamber apical views. We determined left atrial volume (LAV) in systole by biplane area‐length method and report values indexed with body surface area.^26^ Ejection fraction (calculated by the Simpson biplane method), fractional shortening, mitral annular plane systolic excursion (using M‐mode and indexed by left ventricular length), inflows and outflows, and longitudinal tissue velocities as well as strain measurements reflecting left ventricular systolic function were assessed using standard methodology. Left ventricle diastolic function was assessed with LAV, pulsed wave and tissue Doppler, and strain measurements. Right ventricular free wall systolic function was assessed with strain measurements, pulsed wave tissue Doppler, tricuspid annular plane systolic excursion (using M‐mode and indexed by right ventricular length), and right ventricular fractional area change was used as a proxy for right ventricular ejection fraction. Right ventricular diastolic function was assessed with pulsed wave and tissue Doppler as well as strain measurements. Ventricular basal sphericity index was calculated by dividing corresponding ventricle length with basal diameter. Ventricular long‐axis length was defined as the distance from the middle of the mitral/tricuspid valve plane to the corresponding ventricular apex measured at end‐diastole from a standard 4‐chamber view/right ventricular tilted 4‐chamber view. Ventricular base was assessed similarly from the same view at the mitral/tricuspid plane according to pediatric quantitative echocardiography guidelines.^22^

Intervariability coefficients of variation were 2.9% for LVM, 3.6% for LAV, 5.2% for right ventricular basal sphericity index, 3.6% for mitral *E*/*A* ratio, 1.1% for mitral lateral *E′*‐wave peak velocity, and 1.4% for mitral septal *E'*‐wave peak velocity. Intervariability coefficients of variation were for 4‐ and 2‐chamber views' global longitudinal peak systolic strain −9.4% and −5.6%, respectively.

### BP and Pulse Wave Analysis

Office BP was obtained at rest in a sitting position from the nondominant arm with Omron HBP‐1300 and HBP‐1320 devices (Omron Healthcare, Inc., Bannockburn, IL).^27^ Ambulatory BP monitoring was performed with an oscillometric Schiller BR‐102 plus device (Schiller AG, Baar, Switzerland) for 24 hours. Daytime BP was obtained every 30 minutes and at night at 1‐hour intervals, and information on day–night transitions were collected from individual BP diaries.^28^ In accordance with the pediatric ambulatory BP guidelines,^28^ at least 65% of measurements had to be valid, and therefore 16 daytime and 18 nighttime BP registrations were excluded. Central BP values were automatically generated by a pulse wave velocity device (Complior Analyze; Alam Medical, Saint‐Quentin‐Fallavier, France) by using the carotid waveform and office brachial diastolic BP and mean BP for calibration.

### Body Composition and Anthropometrics

Body composition (fat mass, body fat percentage, LBM, and skeletal muscle mass) was obtained with bioelectrical impedance (InBody 720; InBody, South Korea). Body surface area was calculated with Haycock formula. We calculated BMI from body height and weight obtained with the Seca 285 scale and stadiometer (to the closest 0.1 cm and 0.05 kg, respectively; Seca GmBH & Co, Hamburg, Germany). Ponderal index was generated as body weight divided by body height to the third power. The circumferences of the head, thorax, waist, and hip were measured with a tape measure to the closest 0.1 cm.

### Index Pregnancy Data and Questionnaires

The original FINNPEC database contained information on maternal and perinatal data for the index pregnancy.^21^ Preeclampsia was defined as early‐onset or late‐onset on the basis of either diagnosis or delivery earlier than or ≥34^0/7^ gestational weeks, respectively.^29^ Delivery before 37^0/7^ gestational weeks was classified as premature and birth weight <−2 SDs as SGA. Based on national birth data, *Z* scores were generated for birth height, weight, and head circumference.^30^ Current information on family background (eg, diseases), parental smoking and alcohol intake, and annual household income were assessed with standard questionnaires.

### Postnatal Growth

Information on postnatal growth was obtained from primary care growth charts. PE children had available growth data at 6‐month and 1‐year examinations for N=122 (36 early‐onset) and N=119 (35 early‐onset), respectively. Forty‐seven non‐PE children had available growth data at the 6‐month examination and 49 non‐PE children at the 1‐year examination. To rule out possible selection bias, key characteristics were compared at birth and follow‐up between included participants and participants with missing longitudinal growth data. Birth and 11‐year follow‐up characteristics were not different among participants with missing growth data in between (results not shown). Height, weight, BMI, and head circumference *Z* scores were calculated in relation to sex and age, with weight *Z* score also generated for sex and height on the basis of a national population data set.^31^, ^32^ Prematurity was corrected for age until the 3‐year follow‐up. Anthropometric *Z* scores at 3‐ to 5‐week and 2‐month examinations were calculated using gestational age instead of chronological age for premature children <40 gestational weeks.^30^

### Statistical Analysis

Results are presented as mean (SD) or median (interquartile range) for normally or nonnormally distributed numerical data, respectively, and as count (percentage) for categorical data. We checked normality distribution with histograms, normality tests (Kolmogorov–Smirnov and Shapiro–Wilk), Q‐Q plots, and skewness. We evaluated group differences with the independent samples *t* test, Mann–Whitney *U* test, Pearson χ^2^ test or 2‐tailed Fisher's exact test, as appropriate. Only echocardiography variables' mean differences (or median differences) with a *P* value ≤0.010 are reported in the Results section due to multiple testing causing possibility of type 1 error.

Univariate linear regression analyses for potential predictors of children's LVM and LAV are displayed in domains in Table S1: (1) child sex, (2) child age, (3) child anthropometrics, (4) child adiposity, (5) child postnatal growth, (6) office BP, (7) ambulatory BP, and (8) child office central BP. Only associations with a *P* value ≤0.010 are reported in the Results section when considering the number of tests in univariate linear regression analyses and type 1 error possibility.

We built multiple linear regression models to assess the combined influence of preeclampsia, BP, body composition, and other potential predictors on children's LVM and LAV. Predictors were chosen primarily on the basis of prior knowledge as well as univariate linear regression analyses. Models were examined for linearity, normality, and homoscedasticity. Variance influence factor <2.5 and collinearity tolerance >0.3 were deemed appropriate when evaluating models' multicollinearity, and *P* values <0.05 were considered significant when no multiple testing in these analyses. Cross‐validation was done by using different variables for adiposity and BP in the models and obtaining similar results regarding adiposity's and BP's impact on LVM/LAV. Statistical analysis was performed using SPSS version 27 (IBM, Armonk, NY) and 2‐tailed tests.

### Independent Data Access and Analysis

T.S. had access to all study data and takes responsibility for its integrity and analysis.

## Results

### Perinatal and Postnatal Growth and Follow‐Up Child Characteristics

A total of 182 PE (46 early‐onset and 136 late‐onset) and 85 non‐PE children were examined at the follow‐up visit at mean age 11.4 years with no differences in body size or composition (Table 1). No child suffered from congenital heart disease or sleep apnea. Prematurity and SGA were more common in the PE group (including early‐ and late‐onset PE subgroups; Table 1). PE children's birth anthropometrics were significantly smaller than non‐PE children, and this was accentuated in the early‐onset PE group.

**Table 1:** Children's Birth and Follow‐Up Characteristics

	Non‐PE	PE	Early‐onset preeclampsia	Late‐onset preeclampsia	*P* value	Mean difference (95% CI)
			Diagnosis	Diagnosis	PE vs Non‐PE	
			<34^0/7^ weeks	≥34^0/7^ weeks		
Birth characteristics	N=85	N=182	N=46	N=136		
Premature (n, %)	4 (4.7)	60 (33.0)	42 (91.3)	18 (13.2)	<0.001*	…
SGA (n, %)	0 (0)	32 (17.6)	15 (32.6)	17 (12.5)	<0.001*	…
Birth weight, g	3614±54	2769±842	1844±811	3082±584	<0.001*	−845 (−1007 to −683)
Follow‐up characteristics	N= 85	N=182	N=46	N=136		
Age, y	11.2±1.0	11.6±1.1	11.6±1.2	11.6±1.1	0.004*	0.4 (0.1 to 0.7)
Girls (n, %)	42 (49.4)	99 (54.4)	25 (54.3)	74 (54.4)	0.447	…
Body height, cm	149.3±8.4	151.2±10.1	148.7±9.1	152.0±10.3	0.132	1.9 (−0.6 to 4.4)
Body height *Z* score	0.15±0.89	0.05±1.10	−0.30±1.18	0.17±1.06	0.450	−0.10 (−0.37 to 0.17)
Body weight, kg	39.4±10.4±†	40.3±17.1±†	41.2±18.5±†	40.1±16.0†	0.427	1.0 (−1.6 to 3.5)†
Body weight *Z* score, age	0.06±0.96	−0.06±1.06	−0.04±1.21	−0.06±1.01	0.410	−0.11 (−0.38 to 0.15)
Body weight *Z* score, height	−0.13±0.95	−0.22±1.10	0.16±0.97	−0.35±1.11	0.502	−0.09 (−0.36 to 0.18)
BMI, kg/m^2^	17.8±3.1†	17.6±4.1†	18.6±4.7†	17.5±4.0†	0.993	−0.01 (−0.71 to 0.70)†
BMI *Z* score	−0.02±0.97	−0.13±1.08	0.14±1.05	−0.22±1.08	0.443	−0.11 (−0.38 to 0.17)
Body surface area, m^2^ ‡	1.30±0.23	1.33±0.22	1.33±0.24	1.33±0.22	0.228	0.03 (−0.02 to 0.08)
Waist circumference, cm	63.0±8.9†	64.1±11.0†	67.0±12.2†	63.2±10.5†	0.331	1.0 (−1.0 to 3.0)†
Lean mass bioimpedance, kg	32.6±5.7	33.7±7.2	32.9±6.6	33.9±7.4	0.175	1.1 (−0.5 to 2.7)
Skeletal muscle mass, kg	17.2±3.4	17.9±4.3	17.4±3.9	18.0±4.4	0.162	0.7 (−0.3 to 1.6)
Fat mass, kg	6.3±5.7†	6.6±7.1†	7.5±9.1†	6.6±5.6†	0.914	0.1 (−1.0 to 1.1)†
Body fat percentage, %	19.0±8.3	18.9±8.9	21.2±9.6	18.1±8.5	0.920	−0.1 (−2.4 to 2.1)
24‐h blood pressure	N=63	N=144	N=35	N=109		
SBP, mm Hg	119.6±6.8	122.5±8.8	125.1±9.7	121.7±8.4	0.024*	2.9 (0.4 to 5.3)
DBP, mm Hg	71.3±5.4	70.4±5.8	70.2±6.3	70.5±5.6	0.284	−0.9 (−2.6 to 0.8)
PP, mm Hg	48.4±5.2	52.1±7.6	55.1±9.4	51.1±6.7	<0.001*	3.7 (1.9 to 5.4)
HR, bpm	81.6±7.2	80.9±7.7	81.7±7.8	80.6±7.6	0.534	−0.7 (1.1 to −3.0)
SBP *Z* score, height	1.26±0.99	1.60±1.24	2.07±1.29	1.45±1.19	0.058	0.34 (−0.01 to 0.69)
DBP *Z* score, height	0.80±0.99	0.62±1.05	0.60±1.09	0.63±1.04	0.256	−0.18 (−0.49 to 0.13)

Data are presented as mean±SD unless stated otherwise. Independent samples *t* test for normally distributed numerical data, Mann–Whitney *U* test for nonnormal distribution and Pearson χ^2^ test or Fisher's exact test for categorical data. BMI indicates body mass index; DBP, diastolic blood pressure; HR, heart rate; PE, preeclampsia‐exposed; PP, pulse pressure; SBP, systolic blood pressure; and SGA, small for gestational age (birth weight ≤2SD; premature, birth <37+0 gestational weeks).

* Significant *P* value (<0.05).

^†^ Median (interquartile range), median difference (95% CI).

^‡^ Calculated with Haycock's formula.

PE children's postnatal catch‐up growth for weight *Z* score (corrected for age and sex) occurred during the first 6 months from birth (Figure). These findings were accentuated in the early‐onset PE group. Early childhood BMI peaked at 6 months for PE children compared with 1 year for non‐PE children due to catch‐up growth among PE children. Ponderal index peaked at 4 months in early‐onset preeclampsia and at 2 months in late‐onset preeclampsia, while non‐PE children peaked at birth, followed by a decline with age in all groups.

**Figure 1:**
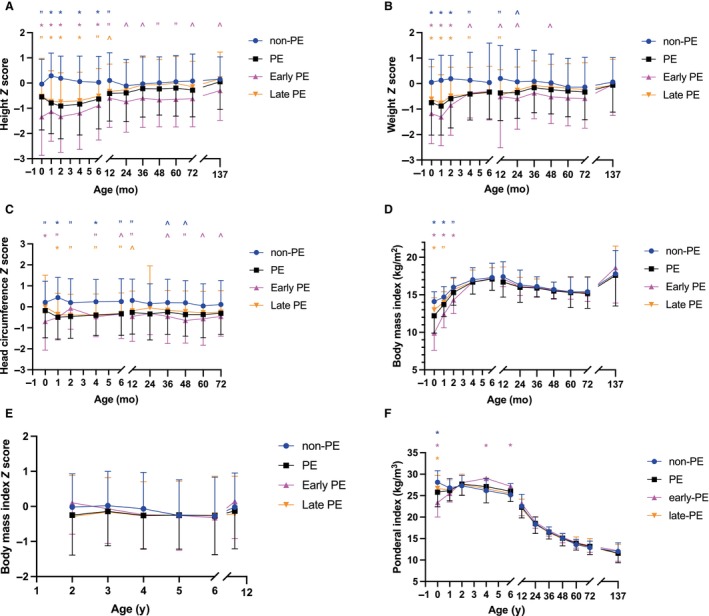
Postnatal growth from birth to 11.4 years of age (mean and SD). **A**, Height *Z* score; (**B**) weight *Z* score (corrected for age and sex); (**C**) head circumference *Z* score; (**D**) body mass index; (**E**) body mass index *Z* score; and (**F**) Ponderal index. Age is displayed on the *x* axis and corrected for prematurity. PE indicates preeclampsia‐exposed group; non‐PE, unexposed control group; early‐PE, maternal preeclampsia diagnosis before 34^0/7^ gestational weeks; late‐PE, maternal preeclampsia diagnosis at or after 34^0/7^ gestational weeks. Significant differences between PE subgroups and non‐PE group are highlighted with *P<0.001, “*P*<0.01, and ^*P*<0.05. Independent samples *t* test for normally distributed numerical data and Mann–Whitney *U* test for nonnormal distribution.

### Cardiac Structure at 8‐ to 12‐Year Follow‐Up

Right ventricular basal sphericity index was significantly lower in PE children (including all subgroups) compared with non‐PE children (mean difference, −0.26 [95% CI, −0.39 to −0.12]; *P*<0.001; Table 2). This difference was accentuated in the early‐onset PE children (mean difference, −0.46 [95% CI, −0.71 to −0.21]; *P*<0.001) and contributed by early‐onset PE children's right ventricular length being significantly shorter than non‐PE children, and right ventricular base borderline wider. Aortic, atrial, and ventricular dimensions, as well as LVM, LVM indexed, and LVM *Z* score were no different between PE and non‐PE children (Table 2, Tables S2 and S3). Early‐ and late‐onset preeclampsia by either diagnosis or delivery definition provided similar results (results not shown).

**Table 2:** Cardiac Structure

	Non‐PE	PE	Early‐onset	Late‐onset	*P* value	Mean difference (95% CI)	*P* value	Mean difference (95% CI)	*P* value	Mean difference (95% CI)
			Diagnosis	Diagnosis	PE vs non‐PE		Early (diagnosis) vs non‐PE		Late (diagnosis) vs non‐PE	
			<34^0/7^ wks	≥34^0/7^ wks						
Left ventricle	N=84	N=176	N=44	N=132						
Interventricular septum dimension, cm	0.56±0.10	0.58±0.10	0.56±0.11	0.58±0.10	0.305	0.01 (−0.01 to 0.04)	0.988	0 (−0.04 to 0.04)	0.192	0.02 (−0.01 to 0.05)
Left ventricular dimension in diastole, cm	4.4±0.4	4.5±0.4	4.5±0.4	4.5±0.4	0.416	0.04 (−0.06 to 0.15)	0.251	0.08 (−0.06 to 0.23)	0.596	0.03 (−0.08 to 0.14)
Left ventricular dimension in systole, cm	3.0±0.3	3.0±0.3	2.9±0.3	3.0±0.3	0.182	−0.05 (−0.14 to 0.03)	0.041	−0.11 (−0.21 to 0)	0.401	−0.04 (−0.12 to 0.05)
Left ventricular posterior wall in diastole, cm	0.60±0.09	0.61±0.10	0.60±0.10	0.61±0.10	0.652	0.01 (−0.02 to 0.03)	0.769	0.01 (−0.03 to 0.04)	0.655	0.01 (−0.02 to 0.03)
Left ventricular mass, g*	75.47±20.24	78.59±22.60	78.69±23.77	78.55±22.29	0.283	3.12 (−2.59 to 8.83)	0.422	3.23 (−4.70 to 11.15)	0.305	3.09 (−2.83 to 9.01)
Left ventricular mass indexed to height, g/m^2.7^	25.45±5.41	25.61±5.82	26.80±6.06	25.21±5.72	0.828	0.16 (−1.32 to 1.65)	0.200	1.35 (−0.73 to 3.43)	0.767	−0.23 (−1.77 to 1.31)
Left ventricular mass *Z* score†	−0.86±0.86	−0.81±0.91	−0.71±0.99	−0.84±0.88	0.675	0.05 (−0.18 to 0.28)	0.393	0.14 (−0.19 to 0.48)	0.883	0.02 (−0.22 to 0.26)
Left ventricular basal sphericity index (no unit)	2.36±0.25	2.38±0.29	2.39±0.28	2.37±0.29	0.592	0.02 (−0.05 to 0.09)	0.468	0.04 (−0.06 to 0.13)	0.709	0.01 (−0.06 to 0.09)
Right ventricle										
Right ventricular length, cm	6.0±0.7‡	5.9±1.3‡	5.3±1.4	5.7±1.2	0.055	−0.20 (−0.40 to 0)‡	0.007§	−0.5 (−0.9 to −0.1)‡	0.221	‐0.1 (−0.4 to 0.1)‡
Right ventricular base, cm	3.0±0.5±‡	3.1±0.9‡	3.2±3.5‡	3.0±0.7‡	0.084	0.10 (0 to 0.30)‡	0.019	0.30 (0 to 0.60)‡	0.253	0.1 (−0.1 to 0.2)‡
Right ventricular basal sphericity index, no unit	2.04±0.41	1.78±0.69	1.58±0.77	1.85±0.65	<0.001§	−0.26 (−0.39 to −0.12)	<0.001§	−0.46 (−0.71 to −0.21)	0.010§	−0.19 (−0.33 to −0.05)
Right atrial area in systole, cm^2^	11.1±2.2	10.8±2.3	10.7±2.3	10.9±2.2	0.384	−0.3 (−0.9 to 0.3)	0.345	−0.4 (−1.2 to 0.4)	0.491	−0.2 (−0.8 to 0.4)
Right atrial area indexed to body surface area, cm^2^/m^2^	8.6±1.4	8.2±1.3	8.2±1.4	8.2±1.3	0.081	−0.32 (−0.69 to 0.04)	0.195	−0.3 (−0.9 to 0.2)	0.106	−0.3 (−0.7 to 0.1)

Data are presented as mean±SD unless stated otherwise. Independent samples *t* test for normally distributed numerical data and Mann–Whitney *U* test for nonnormal distribution. PE indicates preeclampsia‐exposed.

* Calculated with Deveraux formula.

^†^ *Z* score calculated for sex and lean body mass.

^‡^ Median (interquartile range), median difference (95% CI).

^§^ Significant *P* value (≤0.01).

### Cardiac Function at 8‐ to 12‐Year Follow‐Up

No study subject presented with evidence of increased right ventricular pressure as assessed by tricuspid regurgitation velocity, ventricular septal shape, and pulmonary artery systolic pulsed‐wave Doppler (PWD) shape. Mitral lateral *E*′‐wave peak velocity was consistently lower in PE children and all subgroups compared with non‐PE children (mean difference, −1.4 cm/s [95% CI, −2.1 to −0.6]; *P*<0.001; Table 3), and this was also reflected in the *E* to *E*′ ratio (mean difference, 0.40 [95% CI, 0.15–0.65]; *P*=0.002). Global basal circumferential early diastolic strain rate was significantly lower in PE (including early‐ and late‐onset PE subgroups) versus non‐PE children (mean difference, −0.20 [95% CI, −0.33 to −0.07]; *P*=0.002; Table S4). No other consistent differences in left ventricular diastolic function parameters were found (Table 3, Tables S2 and S4). Right ventricular diastolic strain parameters were similar between PE and non‐PE children (Table 3 and Table S4).

**Table 3:** Cardiac Function and Strain

	Non‐PE	PE	Early‐onset	Late‐onset	*P* value	Mean difference (95% CI)	*P* value	Mean difference (95% CI)	*P* value	Mean difference (95% CI)
			Diagnosis	Diagnosis	PE vs Non‐PE		Early (dg) vs non‐PE		Late (dg) vs non‐PE	
			<34^0/7^ weeks	≥34^0/7^ weeks						
Diastolic function	N=84	N=176	N=44	N=132						
Left atrial volume, mL	27.3±9.9	27.0±8.7	27.4±8.7	26.9±8.7	0.802	−0.3 (−2.7 to 2.1)	0.947	0.1 (−3.4 to 3.6)	0.730	−0.4 (−3.0 to 2.1)
Left atrial volume indexed, mL/m^2^ *	21.0±6.5	20.4±5.4	20.9±5.7	20.2±5.3	0.399	−0.7 (−2.2 to 0.9)	0.896	−0.2 (−2.5 to 2.2)	0.317	−0.8 (−2.4 to 0.8)
Mitral *E*‐wave peak velocity, cm/s	93.2±15.9	94.8±17.2	101.5±16.5	92.6±16.8	0.488	1.5 (−2.8 to 5.9)	0.007†	8.3 (2.3 to 14.3)	0.780	−0.6 (−5.2 to 3.9)
Mitral A‐wave peak velocity, cm/s	31.9±16.6‡	33.3±13.8‡	35.1±15.3‡	33.3±12.9‡	0.665	0.5 (−2.0 to 3.0)‡	0.984	0.04 (−3.86 to 3.93)	0.666	0.5 (−2.0 to 3.0)‡
Mitral *E*/*A* ratio (no unit)	2.89±0.95	2.89±0.96	2.74±1.03‡	2.75±1.07‡	0.977	0 (−0.25 to 0.25)	0.343	0.16 (−0.19 to 0.51)‡	0.546	−0.08 (−0.33 to 0.16)‡
Tricuspid E‐wave peak velocity, cm/s	58.4±11.0‡	56.8±9.0‡	55.4±12.0‡	57.4±9.0‡	0.199	−1.5 (−3.7 to 0.8)‡	0.058	−3.20 (−6.30 to 0)‡	0.405	−1.1 (−3.6 to 1.4)
Tricuspid A‐wave peak velocity, cm/s	29.0±8.1	29.8±7.0	29.8±6.6	29.9±7.1	0.436	0.8 (−1.2 to 2.9)	0.597	0.8 (−2.1 to 3.7)	0.466	0.8 (−1.4 to 3.0)
Tricuspid *E*/*A* ratio (no unit)	2.16±0.56	2.03±0.55	1.88±1.00‡	1.98±1.00‡	0.100	−0.13 (−0.28 to 0.03)	0.060	−0.20 (−0.40 to 0.01)	0.166	−0.11 (−0.27 to 0.05)‡
Mitral lateral *E*'‐wave peak velocity, cm/s	22.0±2.8	20.6±3.0	20.3±3.3	20.7±3.0	<0.001†	−1.4 (−2.1 to −0.6)	0.004†	−1.7 (−2.8 to −0.5)	0.002†	−1.3 (−2.1 to −0.5)
*E* to *E*‐prime ratio (no unit)	4.30±0.84	4.70±1.12	5.10±1.14	4.57±1.08	0.002†	0.40 (0.15 to 0.65)	<0.001†	0.80 (0.44 to 1.15)	0.055	0.27 (−0.01 to 0.54)
Mitral septal *E*'‐wave peak velocity, cm/s	14.6±1.8	14.6±1.8	14.5±1.7	14.6±1.7	0.883	0.03 (−0.42 to 0.49)	0.816	−0.1 (−0.7 to 0.6)	0.773	0.1 (−0.4 to 0.6)
*E* to *E*‐prime ratio (no unit)	6.45±1.17	6.57±1.40	7.07±1.36	6.41±1.37	0.514	0.12 (−0.23 to 0.46)	0.009†	0.61 (0.15 to 1.07)	0.797	−0.05 (−0.40 to 0.31)
Systolic function										
Left ventricular fractional shortening, %	31.7±4.4	33.6±4.8	35.4±4.8	33.1±4.7	0.003†	1.9 (0.7 to 3.1)	<0.001†	3.6 (2.0 to 5.3)	0.042	1.31 (0.05 to 2.58)
Left ventricular ejection fraction by Simpson biplane, %	62.5±4.3	62.1±3.8	62.4±3.5	62.0±3.9	0.429	−0.4 (−1.5 to 0.6)	0.886	−0.1 (−1.6 to 1.4)	0.360	−0.5 (−1.6 to 0.6)
Right ventricular fractional area change, %	42.5±7.2	43.6±5.4	44.1±5.9	43.5±5.3	0.205	1.1 (−0.6 to 2.9)	0.218	1.6 (−0.9 to 4.1)	0.281	1.0 (−0.8 to 2.8)
Mitral annular plane systolic excursion, cm	1.52±0.17	1.52±0.20	1.54±0.24	1.51±0.19	0.938	0 (−0.05 to 0.05)	0.490	0.03 (−0.05 to 0.10)	0.819	−0.01 (−0.06 to 0.05)
Mitral annular plane systolic excursion, indexed (no unit)§	0.22±0.02	0.22±0.03	0.22±0.03	0.22±0.03	0.436	0 (−0.01 to 0)	0.751	0 (−0.01 to 0.01)	0.384	0 (−0.01 to 0)
Tricuspid annular plane systolic excursion, cm	2.02±0.27	2.10±0.32	2.18±0.38	2.07±0.29	0.071	0.07 (−0.01 to 0.15)	0.024	0.15 (0.02 to 0.28)	0.230	0.05 (−0.03 to 0.13)
Tricuspid annular plane systolic excursion, indexed (no unit)§	0.33±0‡	0.35±0‡	0.38±0‡	0.35±0‡	0.001†	0.03 (0.01 to 0.05)‡	<0.001†	0.06 (0.03 to 0.11)‡	0.015	0.02 (0 to 0.04)‡
Strain										
Left ventricle	N=75	N=166	N=43	N=123						
Global basal circumferential peak systolic strain, %	−21.5±3.3	−21.3±3.3	−22.0±3.5	−21.0±3.2	0.578	0.3 (−0.6 to 1.2)	0.458	−0.5 (−1.8 to 0.8)	0.278	0.5 (−0.4 to 1.4)
4‐chamber view	N=75	N=167	N=43	N=124						
Global longitudinal peak systolic strain, %	−20.4±2.5	−21.0±2.9	−21.6±3.0	−20.7±2.8	0.176	−0.5 (−1.3 to 0.2)	0.038	−1.1 (−2.2 to −0.1)	0.443	−0.3 (−1.1 to 0.5)
2‐chamber view	N=74	N=167	N=43	N=124						
Global longitudinal peak systolic strain, %	−23.5±3.2	−23.0±3.1	−24.0±2.8	−22.7±3.1	0.338	0.4 (−0.4 to 1.3)	0.375	−0.5 (−1.7 to 0.6)	0.107	0.7 (−0.2 to 1.7)
Right ventricle	N=66	N=157	N=41	N=116						
Free wall global longitudinal peak systolic strain, %	−26.5±5.9	−27.5±4.6	−28.1±4.8	−27.3±4.5	0.239	−1.0 (−2.6 to 0.6)	0.149	−1.6 (−3.8 to 0.6)	0.380	−0.7 (−2.4 to 0.9)

Data are presented as mean±SD unless stated otherwise. Independent samples *t* test for normally distributed numerical data and Mann–Whitney *U* test for nonnormal distribution. PE indicates preeclampsia‐exposed.

* Indexed to body surface area.

^†^ Significant *P* value (≤0.01).

^‡^ Median (interquartile range), median difference (95% CI).

^§^ Divided by corresponding ventricle length.

Left ventricular systolic function was similar in PE compared with non‐PE children (Table 3, Tables S2 and S4). For the right ventricle, tricuspid annular plane systolic excursion indexed was significantly higher in PE (including early‐onset PE) compared with non‐PE children (mean difference, 0.03 [95% CI, 0.01–0.05]; *P*=0.001; Table 3). However, no other significant differences in right ventricular systolic function were found. Early‐ and late‐onset preeclampsia by either diagnosis or delivery definition provided similar results for cardiac function (results not shown).

### Predictors of Cardiac Structure and Function in PE and Non‐PE Children

Univariate regressions examining PE and non‐PE children's potential predictors for LVM and LAV are displayed in Table S1. LVM was associated with child age and all anthropometric and adiposity measures at follow‐up, with LBM and muscle mass showcasing the strongest associations. LVM was also associated with office BPs, office central SBP and pulse pressure (PP), carotid–femoral pulse wave velocity, and 24‐hour SBP, PP, and heart rate (including daytime and nighttime). LVM displayed significant relations with change in weight *Z* score during the first year of life and changes in height and weight *Z* scores from birth to follow‐up. The changes in height and weight *Z* scores further associated significantly with body anthropometrics at the follow‐up visit, including LBM and body weight (results not shown).

Multiple predictors' combined influence on PE and non‐PE children's LVM was evaluated with multiple linear regression models (Table 4). Child LBM, body fat percentage, and 24‐hour SBP at follow‐up were all significant, independent predictors of LVM (model's adjusted *R* ^2^, 0.543; *P*<0.001), and with LBM displaying the highest standardized coefficient (0.663). Child body height at follow‐up performed slightly less than LBM with the model's adjusted *R* ^2^ (0.468). Both child body fat percentage and 24‐hour SBP remained as significant, independent predictors in the LVM model with child height or LBM as the main predictors. Children in the highest body fat percentage or 24‐hour SBP quantile had significantly higher LVM than those in the respective lowest quantile (results not shown). Furthermore, male sex was an independent predictor only in the model without LBM. Preeclampsia exposure was nonsignificant in all LVM models, and child age at follow‐up did not improve models (results not shown). Maternal prepregnancy BMI was the only maternal, gestational, and perinatal factor that showcased associations in univariate analyses but did not remain a significant predictor in any of the LVM models (results not shown).

**Table 4:** Multiple Linear Regression Models for All Children's Cardiac Structure and Function

Vascular dimension		Predictor	Unstandardized	Standardized β	*P* value	Adjusted *R* ^2^	Model *P* value
			β (95% CI)				
Left ventricular mass	Model 1	Constant	−30.6 (−61.0 to −0.2)			0.543	<0.001*
		Preeclampsia (0=no, 1=yes)	−1.1 (−5.6 to 3.4)	−0.024	0.620		
		Child lean body mass at follow‐up, kg	2.0 (1.7 to 2.3)	0.663	<0.001*		
		Child body fat percentage, %	0.4 (0.1 to 0.6)	0.145	0.004*		
		Child 24‐h SBP, mm Hg	0.28 (0.03 to 0.54)	0.109	0.027*		
	Model 2	Constant	−169.6 (−214.3 to −125.0)			0.468	<0.001*
		Preeclampsia (0=no, 1=yes)	−1.0 (−5.9 to 3.8)	−0.022	0.680		
		Child sex (0=female, 1=male)	4.7 (0.2 to 9.1)	0.108	0.039*		
		Child height at follow‐up, cm	1.2 (1.0 to 1.5)	0.569	<0.001*		
		Child body fat percentage, %	0.6 (0.3 to 0.9)	0.232	<0.001*		
		Child 24‐h SBP, mm Hg	0.4 (0.1 to 0.7)	0.152	0.004*		
Left atrial volume	Model 1	Constant	1.0 (−4.2 to 6.3)			0.296	<0.001*
		Preeclampsia (0=no, 1=yes)	−0.6 (−2.8 to 1.5)	−0.032	0.570		
		Child sex (0=female, 1=male)	1.7 (−0.3 to 3.7)	0.095	0.087		
		Child lean body mass at follow‐up, kg	0.6 (0.4 to 0.8)	0.433	<0.001*		
		Child body fat percentage, %	0.2 (0.1 to 0.3)	0.181	0.002*		
		Child office central PP, mm Hg	0.07 (−0.03 to 0.16)	0.079	0.194		

PP indicates pulse pressure; and SBP, systolic blood pressure.

* Significant results are *P* value <0.05.

LAV associated with child age and all anthropometric and adiposity measures at follow‐up, with thoracic circumference, body surface area, LBM, and muscle mass showcasing the strongest associations in univariate regression analyses (Table S1). LAV displayed significant relations with office and central SBP and PP, as well as 24‐hour PP and heart rate (including daytime and nighttime). LAV further associated with change in weight *Z* score from birth to follow‐up, and this weight *Z* score change associated with body anthropometrics at follow‐up, including LBM and body weight (results not shown). There were no significant associations between LAV and maternal, gestational, and perinatal factors (results not shown).

In multiple linear regression analyses, child LBM and body fat percentage were significant, independent predictors of LAV (model's adjusted *R* ^2^, 0.296; *P*<0.001; Table 4). Preeclampsia exposure was nonsignificant, while male sex showcased borderline significance (*P*=0.087). Office central PP was not a significant independent predictor, but improved the model's adjusted *R* ^2^ and was therefore included in the final model. Children in the highest body fat percentage or office central PP quantile had significantly higher LAV than those in the respective lowest quantile (results not shown). Child age at follow‐up did not improve the model (results not shown).

## Discussion

In this study, 8‐ to 12‐year‐old PE children show a more globular‐shaped right ventricle with higher longitudinal systolic displacement than age‐matched non‐PE children. Altered left ventricular diastolic indices are also noted in PE children compared with non‐PE children. These changes in cardiac structure and function are accentuated in the early‐onset PE children. LVM and LAV are similar between PE and non‐PE children, and independently predicted by child LBM and adiposity, and LVM further by 24‐hour SBP. Overall, these adverse relations may reflect adverse remodeling of cardiac structure during growth in preadolescent children with increased adiposity and BP, as LVM has been linked with increased CVD death and morbidity risk in adults.^1^

In the present study, lower right ventricular basal sphericity index was seen in PE children, and particularly in early‐onset preeclampsia. This more globular right ventricular shape was due to shorter right ventricular length and wider right ventricular base. To the best of our knowledge, a globular‐shaped right ventricle is a novel finding in preadolescent PE children. Premature infants have displayed a more globular‐shaped heart at birth but normalization by 3 months of age.^33^ Lewandowski et al reported a distinctive left ventricular shape with shorter length and diameter, increased wall thickness, and displaced apex in preterm born adults.^16^ In a small‐sample‐size preeclampsia study, PE children presented with smaller hearts based on a shorter left ventricular end‐diastolic length.^11^ However, no differences in either cardiac structure or function were reported in a recent retrospective cohort study with 80 PE children and 80 non‐PE children, including early‐ and late‐onset PE subgroup analyses.^10^ Aortic, atrial, and ventricular dimensions; LVM; LVM indexed; and LVM *Z* score were similar between our PE versus non‐PE children. LVM was also similar between 7‐ and 12‐year‐old children born from normotensive pregnancies and children exposed to maternal hypertension in utero in a previous small‐sample‐size study.^34^ No difference in LVM indexed was further noted among 42 PE adolescents in comparison with controls, but increased relative wall thickness and smaller left ventricular end‐diastolic volume were reported.^12^ In the aforementioned prospective cohort study, early changes in maternal gestational SBP were related to increased LVM indexed, left ventricular end‐diastolic volume, and E/A ratio in the offspring.^12^ However, LVM and LAV did not associate with maternal gestational or follow‐up office SBP in our PE and non‐PE children.

Mildly altered left ventricular diastolic indices with lower mitral lateral E'‐wave peak velocity, corresponding higher E to E' ratio, and lower global basal circumferential early diastolic strain rate were noted in our PE children compared with non‐PE children. Possible mild late diastolic dysfunction with increased mitral A‐wave peak velocities was observed in a previous small‐sample‐size study on 5‐ to 8‐year‐old PE children.^11^ To the best of our knowledge, cardiac strain measurements have not been previously reported in PE children. A recent study analyzed a subgroup of adults born prematurely and exposed to preeclampsia in utero, and they displayed even more reduced global longitudinal peak systolic strain than those born prematurely from normotensive pregnancies.^16^ Stage 1 hypertension in adults is associated with lower global longitudinal strain,^35^ and lower global longitudinal strain has been coupled with increased risk of CVD death and morbidity in adulthood.^36^ In the present study, PE children and early‐onset PE children displayed lower global basal circumferential early diastolic strain rate compared with non‐PE children. Taken together, preadolescent PE children display mild alterations in cardiac structure and function, and the present study adds significantly to the previous literature on PE children's cardiac structure and function.

The observed cardiac structural and functional alterations in this study were accentuated in the early‐onset PE children. We have previously reported early‐onset PE children to display the highest BP levels compared with non‐PE children, and this was associated with child prematurity factors and maternal gestational BP.^18^ Our early‐onset PE children were more often born prematurely and SGA and were associated with higher maternal gestational BP values. Early‐ and late‐onset preeclampsia are believed to be of different origins and with different hemodynamics,^37^, ^38^ and the cardiovascular risk profile seems to be different in childhood, in part explained by child prematurity factors and maternal gestational BP. However, LVM and LAV were not independently related to any maternal, gestational, or perinatal factors, including preeclampsia, gestational time, and birth size. Litwin et al likewise found no associations between LVM variables or cardiac function and maternal prepregnancy BMI in 6‐year‐old children exposed to maternal adiposity and gestational diabetes.^39^, ^40^ Studies regarding fetal growth restriction have shown no impact on cardiovascular health during the first 6 months of life^41^ and limited alterations in cardiac structure and function for SGA‐born 5‐ to 6‐year‐old children^17^ and adults.^42^ Nevertheless, LVM in healthy adolescents has also been related to birth weight, but not to early childhood growth.^43^ Our observed LVM and LAV associations with postnatal growth seem to be due to the associations with follow‐up body size parameters. Premature infants have shown postnatally an excessive increase in both left and right ventricular mass in relation to overall body and cardiac size, as well as ventricular dysfunction.^33^ Increased LVM and reduced cardiac function have further been followed into adulthood for those born prematurely.^16^

LBM and body fat percentage were independent predictors of both LVM and LAV and 24‐hour SBP for LVM. LBM as the major predictor of LVM, LAV, and cardiac structures overall is consistent with previous findings in children and adolescents.^17^, ^39^, ^40^, ^44^ Our data then support using LBM to adjust LVM for body size in children,^45^ and both LBM and LVM *Z* score calculators already exist.^24^, ^46^ Litwin et al reported similar associations to ours between LAV and child body fat percentage.^39^, ^40^ However, they did not observe any relations between LVM variables and child body fat percentage in 6‐year‐old children. The present results then indicate that adiposity‐associated remodeling of LVM evolves later in preadolescence. Obesity has been linked with increased LVM in both children and young adults,^5^, ^6^, ^7^, ^8^ as well as with increased left atrial diameter.^47^ Elevated BP and increased BMI already from childhood predict increased LVM and left ventricular hypertrophy in adulthood.^48^ Long‐term exposure to elevated BP from young adulthood has also shown associations with impaired left ventricular function in adults.^49^ We have previously reported elevated BP in 8‐ to 12‐year‐old PE children in comparison with non‐PE children.^18^ The impact of elevated BP already in preadolescent age and its associations with LVM, as well as the impact of adiposity measures, might then suggest early progression of CVD in preadolescent children with risk factors. Adiposity seems to play an important role in pediatric cardiovascular health as adolescents with primary hypertension showcase a decrease in LVM related to a reduction in waist circumference and abdominal adiposity after a 1‐year pharmacological treatment.^50^ The aforementioned study, however, predominantly included males. In the present study, male sex was only an independent predictor of LVM when LBM was replaced with height. This suggests the impact of male sex to be mediated by LBM as supported by previous studies.^17^

Prospective study design, age‐matched control group; large sample size including early‐onset PE children; and extensive assessment of echocardiography, BP, body size and composition, postnatal growth, and maternal, gestational, and perinatal parameters are considered major strengths of this study. Limitations include loss to follow‐up in the original FINNPEC cohort, and data on children's BP and body composition being available only at follow‐up visit 8 to 12 years after the index pregnancy.

To conclude, preadolescent PE children show a more globular‐shaped right ventricle and mildly altered left ventricular diastolic indices, with findings accentuated in early‐onset PE children, in comparison with age‐matched controls without a history of preeclampsia exposure. LVM is strongly predicted by childhood BP, body size, and adiposity, while LAV is predicted by body size and adiposity. Taken together, these associations indicate remodeling of cardiac structure in children with risk factors (increased adiposity and BP) already during a preadolescent age.

## Sources of Funding

The FINNCARE and FINNPEC studies are supported by grants from Academy of Finland; competitive state research funding of the expert responsibility areas of the Helsinki and Uusimaa Hospital and Tampere University Hospital; Dorothea Olivia, Karl Walter, and Jarl Walter Perklén Foundation; Finnish Foundation for Pediatric Research; Jane and Aatos Erkko Foundation; Juho Vainio Foundation; Medicinska stiftelsen i Vasa; Medicinska understödsföreningen Liv och Hälsa; Päivikki and Sakari Sohlberg Foundation; Sigrid Juselius Foundation; and Medical Society of Finland.

## Disclosures

None.

## Supporting information

Tables S1–S4
