# Supplementary material for: Cardiac Structure and Function in 8‐ to 12‐Year‐Old Children Following In‐Utero Exposure to Preeclampsia (FINNCARE Study)
Source: J Am Heart Assoc. 2024 Jul 16;13(15):e034494. doi: 10.1161/JAHA.124.034494 (PMC11964028; doi:10.1161/JAHA.124.034494)
Supplement: Supplementary file 1 — Tables S1–S4 [file JAH3-13-e034494-s001.pdf]

Online Supplementary Material

Cardiac structure and function in 8-12-year-old children following in-utero exposure to preeclampsia (FINNCARE-study)

Michelle Renlund-Vikström, MD[a,b], Tiina J Jääskeläinen, PhD[c,d], Anni Kivelä, MSc[c], Seppo Heinonen, MD, PhD[e], Hannele Laivuori, MD, PhD[c,f], and Taisto Sarkola, MD, PhD[a,b]

[a]Children's Hospital, University of Helsinki and Helsinki University Hospital, Helsinki, Finland
[b]Minerva Foundation Institute for Medical Research, Helsinki, Finland
[c]Medical and Clinical Genetics, University of Helsinki and Helsinki University Hospital, Helsinki, Finland
[d]Department of Food and Nutrition, University of Helsinki, Helsinki, Finland
[e]Department of Obstetrics and Gynecology, Helsinki University Hospital, Helsinki, Finland
[f]Department of Obstetrics and Gynecology, Tampere University Hospital and Tampere University, Faculty of Medicine and Health Technology, Tampere Center for Child, Adolescent, and Maternal Health Research, Tampere, Finland

Address for Correspondence: Michelle Renlund-Vikström, MD, Children's Hospital, Helsinki University Hospital, Stenbäckinkatu 9, POB 281, FIN-00029, Helsinki, Finland.
E-mail address: michelle.renlund@helsinki.fi

SUPPLEMENTARY TABLES

Supplementary Table 1. The results of univariate linear regressions for all children's cardiac structure and function.

	Left ventricular mass				Left atrial volume			
	B (95% CI)	Standardized B	R^2	p	B (95% CI)	Standardized B	R^2	p
Sex (0 = female, 1 = male)	4.6 (-0.6-10.0)	0.107	0.011	0.084	2.3 (0.1-4.5)	0.125	0.016	0.045
Age (years)	8.3 (6.1-10.5)	0.421	0.178	**<0.001**	2.0 (1.0-3.0)	0.246	0.061	**<0.001**
**Anthropometrics**								
Body height (cm)	1.4 (1.2-1.6)	0.607	0.368	**<0.001**	0.4 (0.3-0.5)	0.422	0.178	**<0.001**
Height z-score	8.6 (6.3-11.0)	0.414	0.171	**<0.001**	3.0 (2.0-4.0)	0.342	0.117	**<0.001**
Body weight (kg)	1.3 (1.2-1.5)	0.683	0.467	**<0.001**	0.4 (0.3-0.5)	0.509	0.259	**<0.001**
Weight z-score (height)	8.5 (6.1-10.8)	0.405	0.164	**<0.001**	3.3 (2.3-4.3)	0.380	0.144	**<0.001**
Weight z-score (age)	11.9 (9.7-14.1)	0.560	0.313	**<0.001**	4.2 (3.2-5.1)	0.477	0.228	**<0.001**
Body surface area (m^2)	72.5 (63.4-81.7)	0.696	0.484	**<0.001**	22.3 (17.7-26.8)	0.518	0.268	**<0.001**
Lean body mass (kg)	2.3 (2.0-2.6)	0.701	0.492	**<0.001**	0.7 (0.5-0.8)	0.496	0.246	**<0.001**
Skeletal muscle mass (kg)	3.9 (3.4-4.3)	0.702	0.493	**<0.001**	1.1 (0.9-1.4)	0.490	0.240	**<0.001**
Head circumference (cm)	5.7 (4.2-7.2)	0.427	0.183	**<0.001**	2.0 (1.3-2.6)	0.366	0.134	**<0.001**
Thoracic circumference (cm)	1.9 (1.6-2.2)	0.586	0.343	**<0.001**	0.7 (0.6-0.8)	0.531	0.282	**<0.001**
Waist circumference (cm)	1.3 (1.1-1.5)	0.592	0.351	**<0.001**	0.4 (0.3-0.5)	0.477	0.227	**<0.001**
Hip circumference (cm)	1.5 (1.3-1.7)	0.606	0.367	**<0.001**	0.5 (0.3-0.6)	0.456	0.207	**<0.001**
**Adiposity**								
Waist-hip ratio (no unit)	97.1 (50.2-143.9)	0.246	0.061	**<0.001**	39.3 (19.8-58.7)	0.241	0.058	**<0.001**
BMI (kg/m^2)	3.7 (3.0-4.3)	0.555	0.308	**<0.001**	1.2 (0.9-1.5)	0.436	0.190	**<0.001**
BMI z-score	10.4 (8.2-12.6)	0.498	0.248	**<0.001**	3.9 (2.9-4.8)	0.446	0.199	**<0.001**
Fat mass (kg)	1.7 (1.3-2.1)	0.476	0.227	**<0.001**	0.5 (0.4-0.7)	0.374	0.140	**<0.001**
Fat percentage (%)	0.8 (0.5-1.1)	0.329	0.109	**<0.001**	0.3 (0.2-0.4)	0.294	0.086	**<0.001**
**Postnatal growth**								
Change in height z-score from birth to follow-up	4.2 (2.3-6.2)	0.258	0.066	**<0.001**	1.0 (0.2-1.9)	0.153	0.024	0.014
Change in weight z-score (age) from birth to follow-up	6.6 (4.8-8.4)	0.406	0.165	**<0.001**	1.8 (1.0-2.6)	0.261	9,968	**<0.001**
Change in height z-score from birth to 1 year	2.1 (-0.7-4.8)	0.117	0.014	0.137	-0.1 (-1.1-1.0)	-0.011	0.000	0.887
Change in weight z-score (age) from birth to 1 year	3.5 (1.1-5.9)	0.222	0.049	**0.004**	0.5 (-0.4-1.5)	0.087	0.008	0.270
Change in height z-score from birth to 6 months	-0.2 (-3.0-2.6)	-0.010	0.000	0.897	-0.6 (-1.7-0.5)	-0.085	0.007	0.278
Change in weight z-score (age) from birth to 6 months	1.6 (-0.8-4.1)	0.103	0.011	0.191	0.2 (-0.8-1.2)	0.030	0.001	0.707
BMI peak at 6 months of age (kg/m^2)	1.9 (-0.2-3.9)	0.140	0.020	0.074	1.0 (0.2-1.8)	0.190	0.036	0.015
**Office BP**								
SBP (mmHg)	0.7 (0.5-1.0)	0.314	0.099	**<0.001**	0.16 (0.04-0.27)	0.160	0.026	**0.010**
DBP (mmHg)	0.6 (0.1-1.0)	0.158	0.025	**0.010**	0.01 (-0.17-0.19)	0.005	0.000	0.936
PP (mmHg)	0.7 (0.4-1.1)	0.241	0.058	**<0.001**	0.2 (0.1-0.4)	0.176	0.031	**0.005**
HR (bpm)	-0.2 (-0.4-0.1)	-0.086	0.007	0.174	-0.13 (-0.24- -0.02)	-0.152	0.023	0.017
SBP z-score	2.3 (-1.0-5.6)	0.085	0.007	0.173	-0.01 (-1.38-1.37)	0.000	0.000	0.994
DBP z-score	1.8 (-2.7-6.2)	0.049	0.002	0.431	-0.9 (-2.8-0.9)	-0.063	0.004	0.318
**Ambulatory BP**								
24-hour SBP (mmHg)	0.6 (0.2-0.9)	0.219	0.048	**0.002**	0.12 (-0.03-0.26)	0.110	0.012	0.119
24-hour DBP (mmHg)	-0.5 (-1.0-0.1)	-0.117	0.014	0.097	-0.215 (-0.431-0.002)	-0.137	0.019	0.052
24-hour PP (mmHg)	1.1 (0.7-1.5)	0.346	0.120	**<0.001**	0.3 (0.1-0.4)	0.228	0.052	**0.001**
24-hour HR (bpm)	-0.7 (-1.1- -0.3)	-0.248	0.062	**<0.001**	-0.3 (-0.5- -0.2)	-0.262	0.069	**<0.001**
Daytime SBP (mmHg)	0.5 (0.2-0.8)	0.192	0.037	**0.004**	0.10 (-0.04-0.24)	0.095	0.009	0.162
Daytime DBP (mmHg)	-0.2 (-0.7-0.3)	-0.062	0.004	0.364	-0.1 (-0.3-0.1)	-0.077	0.006	0.256
Daytime PP (mmHg)	0.9 (0.5-1.3)	0.295	0.087	**<0.001**	0.2 (0.1-0.4)	0.186	0.035	**0.006**
Daytime HR (bpm)	-0.7 (-1.1- -0.4)	-0.264	0.070	**<0.001**	-0.3 (-0.4- -0.1)	-0.243	0.059	**<0.001**
Nighttime SBP (mmHg)	0.4 (0.2-0.7)	0.211	0.045	**0.002**	0.07 (-0.05-0.18)	0.078	0.006	0.255
Nighttime DBP (mmHg)	-0.5 (-0.9- -0.1)	-0.167	0.028	0.014	-0.20 (-0.36- -0.04)	-0.169	0.029	0.012
Nighttime PP (mmHg)	1.0 (0.7-1.3)	0.385	0.148	**<0.001**	0.2 (0.1-0.4)	0.250	0.063	**<0.001**
Nighttime HR (bpm)	-0.32 (-0.64- -0.01)	-0.136	0.018	0.045	-0.17 (-0.30- -0.04)	-0.174	0.030	**0.010**
**Central BP and PWV**								
Central SBP (mmHg)	0.7 (0.4-0.9)	0.349	0.122	**<0.001**	0.2 (-0.1-0.3)	0.220	0.048	**<0.001**
Central DBP (mmHg)	0.1 (-0.3-0.6)	0.040	0.002	0.537	-0.1 (-0.3-0.1)	-0.054	0.003	0.402
Central PP (mmHg)	0.7 (0.4-0.9)	0.341	0.116	**<0.001**	0.2 (0.1-0.3)	0.259	0.067	**<0.001**
Carotid-femoral PWV (m/s)	5.7 (2.1-9.3)	0.194	0.038	**0.002**	-0.03 (-1.56-1.51)	-0.002	0.000	0.973

B indicates unstandardized beta; 95% CI, 95% confidence interval; R2, nonadjusted R Square. Significant p-values (≤0.01) bolded.

SBP indicates systolic blood pressure; DBP, diastolic blood pressure; PP, pulse pressure; BMI, body mass index; HR, heart rate; bpm, beats per minute; PWV, pulse wave velocity.

Supplementary Table 2. Cardiac echocardiography parameters

	non-PE	PE	Early-onset *Diagnosis* $<34^{0/7}$ *weeks*	Late-onset *Diagnosis* $\geq 34^{0/7}$ *weeks*	P-value PE vs non-PE	Mean difference (95% CI)	P-value Early (dg) vs non-PE	Mean difference (95% CI)	P-value Late (dg) vs non-PE	Mean difference (95% CI)
**Cardiac four chamber**	*N = 84*	*N = 176*	*N = 44*	*N = 132*						
Left ventricular length diastole (cm)	6.8 (0.5)	6.9 (0.6)	7.0 (0.6)	6.9 (0.7)	0.124	0.11 (-0.03-0.26)	0.123	0.16 (-0.04-0.36)	0.212	0.1 (-0.1-0.3)
Left ventricular base (cm)	2.9 (0.3)	2.9 (0.3)	2.9 (0.3)	2.9 (0.3)	0.465	0.03 (-0.05-0.11)	0.609	0.03 (-0.08-0.14)	0.476	0.03 (-0.06-0.12)
Left ventricular diastolic area (cm^2)	23.9 (3.4)	24.3 (3.8)	24.5 (3.8)	24.3 (3.8)	0.368	0.4 (-0.5-1.4)	0.393	0.6 (-0.7-1.9)	0.436	0.4 (-0.6-1.4)
Left ventricular systolic area (cm^2)	13.0 (2.5)	13.4 (2.5)	13.5 (2.3)	13.4 (2.5)	0.250	0.4 (-0.3-1.0)	0.336	0.4 (-0.5-1.3)	0.306	0.4 (-0.3-1.0)
Left ventricular length systole (cm)	5.3 (0.5)	5.5 (0.6)	5.5 (0.6)	5.5 (0.7)	0.050	0.14 (0-0.29)	0.106	0.16 (-0.03-0.35)	0.083	0.08 (-0.02-0.29)
Left atrial length (cm)	4.3 (0.5)	4.2 (0.5)	4.2 (0.6)	4.2 (0.5)	0.665	-0.03 (-0.17-0.11)	0.726	-0.04 (-0.23-0.16)	0.694	-0.03 (-0.18-0.12)
Left atrial area (cm^2)	11.2 (2.5)	11.0 (2.2)	11.1 (2.4)	11.0 (2.2)	0.585	-0.2 (-0.8-0.4)	0.847	-0.1 (-1.0-0.8)	0.545	-0.2 (-0.8-0.4)
Right ventricle anterior wall thickness in diastole (cm)	0.38 (0.05)	0.40 (0.07)	0.39 (0.08)	0.40 (0.07)	0.035	0.02 (0-0.04)	0.397	0.01 (-0.02-0.04)	0.017	0.02 (0-0.04)
Right ventricular diastolic area (cm^2)	14.0 (2.3)	14.4 (2.9)	14.8 (3.0)	14.2 (2.8)	0.324	0.3 (-0.3-1.0)	0.096	0.8 (-0.1-1.7)	0.632	0.2 (-0.5-0.9)
Right ventricular systolic area (cm^2)	8.1 (1.6)	8.1 (1.8)	8.3 (1.7)	8.0 (1.8)	0.900	0.03 (-0.42-0.48)	0.481	0.2 (-0.4-0.8)	0.891	-0.3 (-0.51-0.44)
**Cardiac function**										
Mitral lateral A'-wave peak velocity (cm/s)	7.4 (1.7)	7.3 (1.7)	7.2 (1.7)	7.4 (1.7)	0.710	-0.1 (-0.5-0.4)	0.552	-0.2 (-0.8-0.4)	0.835	-0.05 (-0.52-0.42)
Mitral septal A'-wave peak velocity (cm/s)	6.1 (1.2)	5.9 (1.1)	5.8 (0.9)	6.0 (1.2)	0.253	-0.2 (-0.5-0.1)	0.136	-0.3 (-0.6-0.1)	0.402	-0.1 (-0.5-0.2)
Tricuspid septal E'-wave peak velocity (cm/s)	15.4 (2.4)	15.3 (2.5)	15.1 (2.5)	15.4 (2.5)	0.807	-0.1 (-0.8-0.6)	0.552	-0.3 (-1.2-0.7)	0.962	-0.02 (-0.73-0.69)
Tricuspid septal A'-wave peak velocity (cm/s)	8.7 (2.1)	8.5 (2.1)	8.4 (1.9)	8.5 (2.1)	0.543	-0.2 (-0.8-0.4)	0.535	-0.2 (-1.0-0.5)	0.623	-0.2 (-0.8-0.5)
Mitral lateral S'-wave peak velocity (cm/s)	10.9 (1.8)	10.5 (1.7)	10.0 (1.6)	10.7 (1.8)	0.068	-0.43 (-0.90-0.03)	**0.005**	-0.9 (-1.6- -0.3)	0.294	-0.3 (-0.8-0.2)
Mitral septal S'-wave peak velocity (cm/s)	8.3 (1.0)	8.1 (1.0)	8.0 (1.0)	8.1 (1.0)	0.068	-0.24 (-0.50-0.02)	0.156	-0.3 (-0.6-0.1)	0.093	-0.23 (-0.51-0.04)
Tricuspid septal S'-wave peak velocity (cm/s)	12.7 (1.4)	12.5 (1.6)	12.2 (1.4)	12.6 (1.7)	0.442	-0.2 (-0.6-0.3)	0.082	-0.5 (-1.0-0.1)	0.781	-0.1 (-0.5-0.4)
Left ventricle end-diastole volume by Simpson biplane (ml)	73.4 (15.2)	75.9 (17.4)	76.2 (17.2)	75.8 (17.5)	0.266	2.5 (-1.9-6.8)	0.353	2.8 (-3.1-8.6)	0.308	2.4 (-2.2-7.0)
Left ventricle end-systolic volume by Simpson biplane (ml)	27.7 (7.4)	28.9 (7.8)	28.7 (7.3)	29.0 (8.1)	0.222	1.2 (-0.8-3.3)	0.433	1.1 (-1.6-3.8)	0.231	1.3 (-0.8-3.5)

Data is presented as mean (SD) unless stated otherwise; significant p-values (≤0.05) bolded. Independent Samples t Test for normally distributed numerical data.

PE indicates preeclampsia; SD, standard deviation; CI, confidence interval; dg, diagnosis.

Supplementary Table 3. Aortic, pulmonary valve and inferior vena cava parameters

	non-PE	PE	Early-onset *Diagnosis* <$34^{0/7}$ *weeks*	Late-onset *Diagnosis* ≥$34^{0/7}$ *weeks*	P-value PE vs non-PE	Mean difference (95% CI)	P-value Early (dg) vs non-PE	Mean difference (95% CI)	P-value Late (dg) vs non-PE	Mean difference (95% CI)
**Aortic structure**										
Aortic valve dimension (cm)	1.68 (0.19)	1.65 (0.19)	1.65 (0.16)	1.66 (0.20)	0.350	-0.02 (-0.07-0.03)	0.337	-0.03 (-0.10-0.03)	0.448	-0.02 (-0.07-0.03)
Aortic root diameter (cm)	2.36 (0.24)	2.31 (0.25)	2.32 (0.23)	2.31 (0.26)	0.131	-0.05 (-0.12-0.02)	0.314	-0.04 (-0.13-0.04)	0.146	-0.05 (-0.12-0.02)
Aortic sinotubular junction (cm)	2.00 (0.23)	1.97 (0.22)	1.98 (0.20)	1.97 (0.22)	0.382	-0.03 (-0.08-0.03)	0.575	-0.02 (-0.11-0.06)	0.399	-0.03 (-0.09-0.04)
Aortic proximal arch diameter (cm)	1.93 (0.26)	1.96 (0.33)	1.95 (0.31)	1.97 (0.34)	0.335	0.04 (-0.04-0.11)	0.619	0.03 (-0.08-0.13)	0.329	0.04 (--0.04-0.12)
Aortic distal arch diameter (cm)	1.57 (0.25)	1.55 (0.23)	1.55 (0.21)	1.55 (0.23)	0.543	-0.02 (-0.08-0.04)	0.579	-0.02 (-0.11-0.06)	0.610	-0.02 (-0.08-0.05)
Aortic arch isthmus diameter (cm)	1.44 (0.28)	1.44 (0.23)	1.46 (0.19)	1.43 (0.25)	0.943	0 (-0.07-0.06)	0.677	0.02 (-0.07-0.11)	0.786	-0.01 (-0.08-0.06)
**Abdominal aortic compliance and stiffness**										
Abdominal aortic compliance (%/10mmHg)	7.31 (2.06)	7.02 (2.25)	7.63 (2.39)	6.81 (2.17)	0.327	-0.29 (-0.86-0.29)	0.432	0.32 (-0.49-1.13)	0.100	-0.50 (-1.09-0.10)
Abdominal aortic stiffness index (no unit)	13.37 (3.33)	13.55 (4.00)	12.61 (3.63)	13.87 (4.08)	0.705	0.18 (-0.76-1.12)	0.240	-0.76 (-2.03-0.51)	0.330	0.50 (-0.51-1.51)
Abdominal aortic pulse wave velocity with Doppler (m/s)	4.78 (0.53)	4.80 (0.55)	4.71 (0.45)	4.83 (0.57)	0.835	0.02 (-0.14-0.17)	0.464	-0.07 (-0.27-0.12)	0.576	0.05 (-0.12-0.22)
**Other measures**	*N = 74*	*N = 166*	*N = 43*	*N = 123*						
Aorta ascendens (cm)	2.18 (0.25)	2.11 (0.25)	2.08 (0.25)	2.12 (0.25)	0.052	-0.07 (-0.14-0)	0.049	-0.10 (-0.20-0)	0.112	-0.06 (-0.13-0.01)
Pulmonary valve dimension (cm)	2.64 (0.38)	2.65 (0.32)	2.69 (0.34)	2.63 (0.32)	0.922	0 (-0.09-0.10)	0.527	0.05 (-0.11-0.22)	0.895	-0.01 (-0.11-0.10)
Inferior vena cava lumen diameter resipratory change (%)	61.0 (20.1)	66.8 (16.3)	64.4 (15.4)	67.6 (16.6)	0.029	5.8 (0.6-11.1)	0.360	3.4 (-4.0-10.9)	0.014	6.6 (1.3-11.9)
Abdominal aorta lumen dimension in end-diastole (cm)	1.03 (0.13)	1.03 (0.13)	1.01 (0.15)	1.03 (0.13)	0.598	-0.01 (-0.04-0.03)	0.373	-0.02 (-0.07-0.03)	0.795	-0.01 (-0.04-0.03)
Abdominal aorta lumen dimension in peak systole (cm)	1.33 (0.16)	1.33 (0.15)	1.33 (0.19)	1.33 (0.14)	0.787	-0.01 (-0.05-0.04)	0.900	0 (-0.07-0.06)	0.768	-0.01 (-0.05-0.04)

Data is presented as mean (SD) unless stated otherwise; significant p-values (≤0.05) bolded. Independent Samples t Test for normally distributed numerical data.

PE indicates preeclampsia; SD, standard deviation; CI, confidence interval; dg, diagnosis.

Supplementary Table 4. Cardiac strain rate parameters

	non-PE	PE	Early-onset *Diagnosis* *<34 $^{0/7}$ weeks*	Late-onset *Diagnosis* *≥34 $^{0/7}$ weeks*	P-value PE vs non-PE	Mean difference (95% CI)	P-value Early (dg) vs non-PE	Mean difference (95% CI)	P-value Late (dg) vs non-PE	Mean difference (95% CI)
**Cardiac strain rate**										
Left ventricle										
Global basal circumferential systolic strain rate (1/s)	-1.35 (0.22)	-1.37 (0.21)	-1.38 (0.21)	-1.36 (0.22)	0.598	-0.02 (-0.08-0.04)	0.428	-0.03 (-0.12-0.05)	0.757	-0.01 (-0.07-0.05)
Global basal circumferential early diastolic strain rate (1/s)	2.05 (0.46)	1.85 (0.47)	1.78 (0.43)	1.87 (0.48)	**0.002**	-0.20 (-0.33- -0.07)	**0.002**	-0.27 (-0.44- -0.10)	0.011	-0.18 (-0.31- -0.04)
Global basal circumferential late diastolic strain rate (1/s)	0.44 (0.18)	0.44 (0.16)	0.41 (0.23)*	0.43 (0.19)*	0.844	0 (-0.04-0.05)	0.483	-0.02 (-0.08-0.04)*	0.347	0.02 (-0.02-0.07)*
Four chamber view										
Global longitudinal systolic strain rate (1/s)	-1.17 (0.22)	-1.19 (0.19)	-1.21 (0.21)	-1.19 (0.19)	0.498	-0.02 (-0.07-0.04)	0.377	-0.04 (-0.12-0.04)	0.667	-0.01 (-0.07-0.05)
Global longitudinal early diastolic strain rate (1/s)	2.23 (0.44)	2.35 (0.53)	2.36 (0.51)	2.35 (0.54)	0.062	0.12 (-0.01-0.25)	0.158	0.13 (-0.05-0.30)	0.086	0.12 (-0.02-0.26)
Global longitudinal late diastolic strain rate (1/s)	0.49 (0.20)*	0.50 (0.17)*	0.50 (0.11)	0.51 (0.16)	0.757	0.01 (-0.03-0.05)*	0.797	0.01 (-0.05-0.06)*	0.780	0.01 (-0.04-0.05)*
Two chamber view										
Global longitudinal systolic strain rate (1/s)	-1.29 (0.22)	-1.26 (0.19)	-1.27 (0.18)	-1.25 (0.20)	0.290	0.03 (-0.03-0.09)	0.699	0.02 (-0.06-0.09)	0.253	0.04 (-0.03-0.10)
Global longitudinal early diastolic strain rate (1/s)	2.16 (0.46)	2.14 (0.42)	2.18 (0.36)	2.13 (0.44)	0.734	-0.02 (-0.14-0.10)	0.844	0.02 (-0.15-0.18)	0.613	-0.03 (-0.16-0.10)
Global longitudinal late diastolic strain rate (1/s)	0.46 (0.15)	0.44 (0.14)	0.43 (0.13)	0.44 (0.14)	0.204	-0.03 (-0.06-0.01)	0.264	-0.03 (-0.08-0.02)	0.273	-0.02 (-0.06-0.02)
Right ventricle										
Free wall global longitudinal systolic strain rate (1/s)	-1.64 (0.52)*	-1.63 (0.50)*	-1.67 (0.47)*	-1.73 (0.47)*	0.140	-0.08 (-0.19-0.03)*	0.077	-0.15 (-0.29-0.01)*	0.275	-0.06 (-0.17-0.05)*
Free wall global longitudinal early diastolic strain rate (1/s)	2.07 (0.80)*	2.17 (0.79)*	2.25 (0.64)*	2.15 (0.88)*	0.653	0.04 (-0.15-0.22)*	0.365	0.10 (-0.15-0.33)*	0.870	0.02 (-0.18-0.21)*
Free wall global longitudinal late diastolic strain rate (1/s)	0.90 (0.43)	0.90 (0.41)	0.98 (0.38)	0.88 (0.43)	0.908	0.01 (-0.11-0.13)	0.309	0.08 (-0.08-0.24)	0.764	-0.02 (-0.15-0.11)

Data is presented as mean (SD) unless stated otherwise; significant p-values (≤0.05) bolded. Independent Samples t Test for normally distributed numerical data and Mann-Whitney U Test for non-normal distribution

PE indicates preeclampsia; SD, standard deviation; IQR, interquartile range; CI, confidence interval; dg, diagnosis.

*median (IQR), median difference (95% CI)
